# A Web-Based Self-help Intervention for Coping With the Loss of a Partner: Protocol for Randomized Controlled Trials in 3 Countries

**DOI:** 10.2196/37827

**Published:** 2022-11-30

**Authors:** Jeannette Brodbeck, Sofia Jacinto, Afonso Gouveia, Nuno Mendonça, Sarah Madörin, Lena Brandl, Lotte Schokking, Ana Maria Rodrigues, Judite Gonçalves, Bettina Mooser, Marta M. Marques, Joana Isaac, Vasco Nogueira, Ana Matos Pires, Lex van Velsen

**Affiliations:** 1 School of Social Work University of Applied Sciences and Arts Northwestern Switzerland Olten Switzerland; 2 Department of Clinical Psychology and Psychotherapy University of Bern Bern Switzerland; 3 Centro de Investigação e Intervenção Social (CIS-Iscte) Instituto Universitário de Lisboa (Iscte-IUL) Lisbon Portugal; 4 Psychiatry Department Local Health Unit of Baixo Alentejo (ULSBA) Beja Portugal; 5 Comprehensive Health Research Centre (CHRC) NOVA Medical School, Universidade Nova de Lisboa (NMS/UNL) Lisboa Portugal; 6 EpiDoC Unit, Centro de Estudos de Doenças Crónicas (CEDOC) NOVA Medical School, Universidade Nova de Lisboa (NMS/UNL) Lisboa Portugal; 7 eHealth group Roessingh Research and Development Enschede Netherlands; 8 Research Group of Communication Science University of Twente Enschede Netherlands; 9 The National Foundation for Elderly Amersfoort Netherlands; 10 Nova School of Business and Economics Carcavelos Portugal; 11 NOVA Medical School, Nova University of Lisbon Lisboa Portugal

**Keywords:** bereavement, cognitive behavioral therapy, CBT, cost-effectiveness, electronic mental health, grief, technology acceptance

## Abstract

**Background:**

The death of a partner is a critical life event in later life, which requires grief work as well as the development of a new perspective for the future. Cognitive behavioral web-based self-help interventions for coping with prolonged grief have established their efficacy in decreasing symptoms of grief, depression, and loneliness. However, no study has tested the efficacy for reducing grief after losses occurring less than 6 months ago and the role of self-tailoring of the content.

**Objective:**

This study aims to evaluate the clinical efficacy and acceptance of a web-based self-help intervention to support the grief process of older adults who have lost their partner. It will compare the outcomes, adherence, and working alliance in a standardized format with those in a self-tailored delivery format and investigate the effects of age, time since loss, and severity of grief at baseline as predictors. Focus groups to understand user experience and a cost-effectiveness analysis will complement the study.

**Methods:**

The study includes 3 different randomized control trials. The trial in Switzerland comprises a waitlist control group and 2 active arms consisting of 2 delivery formats, standardized and self-tailored. In the Netherlands and in Portugal, the trials follow a 2-arm design that will be, respectively, complemented with focus groups on technology acceptance and cost-effectiveness analysis. The main target group will consist of adults aged >60 years from the general population in Switzerland (n≥85), the Netherlands (n≥40), and Portugal (n≥80) who lost their partner and seek help for coping with grief symptoms, psychological distress, and adaptation problems in daily life. The trials will test the intervention’s clinical efficacy for reducing grief (primary outcome) and depression symptoms and loneliness (secondary outcomes) after the intervention. Measurements will take place at baseline (week 0), after the intervention (week 10), and at follow-up (week 20).

**Results:**

The trials started in March 2022 and are expected to end in December 2022 or when the needed sample size is achieved. The first results are expected by January 2023.

**Conclusions:**

The trials will provide insights into the efficacy and acceptance of a web-based self-help intervention among older adults who have recently lost a partner. Results will extend the knowledge on the role of self-tailoring, working alliance, and satisfaction in the effects of the intervention. Finally, the study will suggest adaptations to improve the acceptance of web-based self-help interventions for older mourners and explore the cost-effectiveness of this intervention. Limitations include a self-selective sample and the lack of cross-cultural comparisons.

**Trial Registration:**

Switzerland: ClinicalTrials.gov NCT05280041; https://clinicaltrials.gov/ct2/show/NCT05280041; Portugal: ClinicalTrials.gov NCT05156346; https://clinicaltrials.gov/ct2/show/NCT05156346

**International Registered Report Identifier (IRRID):**

PRR1-10.2196/37827

## Introduction

### Background

The death of a partner is a very stressful critical life event in later life. It involves a dissolution of social and emotional ties, which requires the acceptance of the loss as well as the development of a new identity and a new perspective for the future. It involves the adaptation of daily routines, which can be even more challenging when social, physical, and financial resources decrease in later life [1]. Grief and psychological distress after the loss of a partner are normative reactions. For most individuals, grief intensity weakens to a manageable degree within several weeks or months after the loss (eg, [2,3]). However, some individuals are less able to cope with bereavement and show symptoms of prolonged grief or adaptation problems [4-7]. Approximately 10% of mourners develop prolonged grief, which is a psychological disorder characterized by separation distress; frequent or disabling cognitive, emotional, and behavioral symptoms such as avoidance of reminders of the loved one; difficulties moving on with life; and functional impairment [8-10]. A recent study comparing the prevalence of prolonged grief disorder based on 2 criteria sets, Diagnostic and Statistical Manual of Mental Disorders, Fifth Edition, American Psychiatric Association [11] and International Statistical Classification of Diseases and Related Health Problems, 11th Edition, World Health Organization, 2019 [12], found a lower prevalence of the disorder (4.4% and 3.3%, respectively) than the previous studies. However, this study highlighted a higher prevalence of symptoms: difficulty accepting the loss was the most frequent single symptom (14%-25%), and grief-related impairment was common (10%-16%). Crucially, this study showed that >60% of participants with probably prolonged grief disorder used health care services [13]. Therefore, supporting mourners and promoting a healthy grief process is key to preventing further health problems.

### Web-Based Grief Interventions

Several web-based interventions have been developed as a complement to face-to-face grief counseling or therapy [14-18]. A recent meta-analysis, while summarizing the evidence for web-based interventions for mourners, found that all the cognitive behavioral therapy (CBT)–based interventions showed moderate effects (*g*=0.54) for symptoms of grief and large effects (*g*=0.86) for posttraumatic stress disorder, with effects being stable over time [19]. Furthermore, an additional systematic review and meta-analysis with similar aims managed to replicate said results, showing that all 9 assessed randomized controlled trials decreased symptoms of grief after bereavement [20]. However, this meta-analysis clarified that most of the interventions assessed focused on the loss of a child. Only 2 interventions focused on the loss of the partner and on prolonged grief [14,15]. From these, only 1 study aimed at supporting the grief process of older adults [14]. This suggests that the bereavement of older adults when they lose their partners is overlooked, especially considering the potential for developing prolonged grief and other symptoms. Indeed, older adults seem to be underrepresented in web-based CBT interventions in general, as suggested by a meta-analysis testing whether computerized CBT treatments are potential tools to decrease depression in later life [21]. Despite being pervious to challenges related to low digital literacy, web-based interventions aimed at older adults present several benefits such as high accessibility, flexibility, and privacy as well as low costs [22]. Such benefits are expected to outweigh these potential barriers.

CBT techniques such as exposure, cognitive reappraisal, as well as integration and restoration as treatment components have been demonstrated to have good results in web-based self-help interventions for complicated grief after bereavement [17,18]. Furthermore, a study compared exposure on the web with behavioral activation treatment [15], showing that both interventions reduced complicated grief, posttraumatic stress, and grief rumination, but only exposure showed an effect on depression and brooding levels relative to the control group. Most web-based interventions provide standardized modules in a given order. However, the need to achieve better outcomes by adapting treatment planning to each individual has been guiding research in face-to-face CBT interventions (eg, [23]). It also fostered the development of modular intervention formats, which proved to be effective (eg, [24,25]). Rather than following a standardized fixed order, in this type of intervention, modules are implemented according to individuals’ specific symptoms and specific needs at each time (eg, [25]). Following this principle, self-tailored, web-based CBT interventions (iCBT) may lead to promising results as modular face-to-face approaches. A study addressing comorbidity in depression compared self-guided iCBT with standardized (nontailored) iCBT and revealed that both delivery formats of the intervention improved measures of depression, anxiety, and quality of life. This study also revealed that the self-tailored treatment was more effective than the standardized treatment among participants with more severe symptoms at baseline and more comorbidity [26]. However, most of the research tends to compare web-based interventions (regardless of being self-tailored or standardized) with other therapeutic formats, such as care-as-usual or discussion groups, as it is described in a meta-analysis testing the role of self-tailoring in web-based interventions [27].

### The Web-Based Self-help Intervention

The development and evaluation of the web-based self-help intervention for mourners is embedded in the Optimizing the Mental Health and Resilience of Older Adults That Have Lost Their Spouse via Blended, Online Therapy (LEAVES) project [28], a European project, funded by the Active and Assisted Living Programme focused on the development of a web-based self-help program that supports older adults dealing with the loss of their partner and with adapting to the new life situation. The web-based self-help intervention is based on 2 theoretical models describing crucial factors for an adaptive adjustment to bereavement: the 4 tasks of mourning [29] and the dual-process model of coping with bereavement [30,31]. It follows the most relevant CBT elements that cognitive behavioral interventions for prolonged grief are often based on (1) exposure (eg, telling the story of the loss), (2) cognitive reappraisal or restructuring of individual dysfunctional thoughts (eg, guilt or anger) associated with the loss, (3) integration and restoration including self-care and social re-engagement, and (4) behavioral activation [32,33]. As described earlier, previous web-based grief interventions have been testing the benefits of these elements (eg, [15]), although these studies did not combine the 4 elements in a single intervention, as it is the aim of this web-based self-help program. The content of the intervention is based on the LIVIA self-help program for coping with prolonged grief after the loss of a spouse [14,34,35]. LIVIA, as a text-based intervention on the web, without sophisticated interaction with users, proved its efficacy for mourning older adults from a general Swiss population sample with a mean age of 59 years, who had lost their partner at least 6 months ago. It confirmed that the intervention is also efficacious for milder grief symptoms and thus may prevent grief-related disorders. Compared with the waitlist control group, the intervention resulted in significant reductions in grief (Cohen *d*=0.81), depression (Cohen *d*=0.59), and psychopathological distress (Cohen *d*=0.39) as primary outcomes and reductions in embitterment (Cohen *d*=0.37) and loneliness (Cohen *d*=0.37) and an increase in life satisfaction (Cohen *d*=−0.41) as secondary outcomes. These gains were maintained over 3 months. Improvements were similar among participants with low, medium, or high levels of grief at baseline.

The content of the LIVIA program, based on the elements of exposure, cognitive reappraisal, and restoration, has been complemented with an Activities section for behavioral activation, as well as a conversational virtual agent that guides users through the self-help program. In distinct modules, the program provides users the opportunity to cope with their grief in a thorough process from confrontation with the loss to practicing new coping strategies and routines focused on the adaptation to a new life.

### Research Goals

This study has five research goals: (1) to test the efficacy of the web-based self-help program in 3 countries, which includes comparing the active arms with a control group; (2) to examine which delivery format (standardized vs self-tailored) leads to better clinical outcomes; (3) to investigate the mechanisms of change underlying the intervention effects and to examine the roles of predictor variables and mediators; (4) to assess technology acceptance from the perspective of older adults in grief; and (5) to estimate the cost-effectiveness of the web-based service for providers to support the business models and marketing strategies during the implementation of the service. Thus, it provides a comprehensive evaluation of the web-based self-help program.

### Main Research Hypotheses

The main research hypotheses include the following:

1. We hypothesize that the intervention will significantly decrease grief symptoms (primary outcome) as well as depression symptoms and perceived loneliness (secondary outcomes) compared with the control group.
2. Considering that the self-tailored delivery format provides participants with the opportunity to meet their specific needs at any time, we hypothesize that the self-tailored intervention will lead to higher satisfaction with the intervention and to a higher decrease in grief, depression symptoms, and perceived loneliness than the standardized intervention.
3. We hypothesize that the overall helpfulness of the modules, gains in mastery experiences, self-esteem, insights, the matching of the needs of the user (session outcomes), and a better working alliance mediate the effect of the treatment.
4. Comparing the 2 active arms, we hypothesize that the self-tailored intervention will lead to better session ratings and a higher working alliance than the standardized intervention, thus leading to better outcomes after the intervention.

To better understand the intervention effects, exploratory analyses will test whether age, time since loss, and the severity of grief symptoms affect treatment outcomes. Technology acceptance and cost-effectiveness analysis are exploratory and have no a priori hypotheses.

## Methods

### Study Paradigm

The intervention’s efficacy will be tested in Switzerland, Portugal, and the Netherlands, in 3 different trials. Besides the main goal of testing efficacy, each trial will allow meeting different complementary research goals and, consequently, will follow different designs. In the study conducted in Switzerland, the complementary goal will consist of a thorough efficacy evaluation, including the comparison of a standardized with a self-tailored intervention. It consists of a 3-arm randomized controlled trial with 2 active arms (standardized vs self-tailored) and a waitlist control arm and will explore clinical mediators and predictors of the intervention’s effect. Following the principle of parsimony, the trials in the Netherlands and Portugal will follow variations of the main clinical design using a 2-arm randomized controlled trial comparing the intervention with a control group; whereas in the Netherlands trial, whose complementary research goal is to understand technology acceptance, the self-tailored intervention will be compared with a waitlist group. The focus on technology acceptance will be implemented through the conduct of focus groups. In Portugal, where the complementary goal will be to conduct a cost-effective analysis, the study will compare the standardized intervention with a care-as-usual group. The study in Portugal will also include qualitative methodology for the assessment of the barriers to and facilitators of using the web-based self-help program. Testing the web-based self-help intervention in 3 countries is not intended to guide cross-cultural comparisons but to inform different complementary research goals. The web-based intervention will be named differently in the 3 countries: SOLENA in Switzerland, EuLuto in Portugal, and LEAVES in the Netherlands.

### Participants

The main target group is older mourners from the general population (adults aged >60 years) who have lost their partner and seek help in their grief process. Specific inclusion criteria are (1) experience of partner bereavement; (2) seeking help or willingness to accept help to cope with grief symptoms, psychological distress, or the psychosocial adaptation to a life without the deceased; (3) access to an internet connection and adequate equipment; (4) mastery of the country’s first language; and (5) an informed consent by the participant. General exclusion criteria are (1) that the loss occurred <1 month before enrolling in the study, (2) severe psychological or somatic disorders that need immediate treatment and hinder the continuous work on the web-based self-help program, (3) acute suicidality, (4) no emergency plan that specifies a health care professional or service who participants can turn to if they find themselves in crisis, and (5) cognitive or physical inability to follow the procedures of the study. Considering that this research does not have an age-related hypothesis, the trials do not impose an age limit for the participants.

### Sample Size

Sample size was determined by a power analysis based on a probability level of 0.05 and a power of 0.80 with G*Power [36], which is based on the results of the evaluation of LIVIA [14]. As in LIVIA, we expected a large effect size of Cohen *d*>0.80 for the decrease in grief as primary outcome, measured by the Texas Revised Inventory of Grief (TRIG), in the comparison of the intervention with a waitlist control group or care-as-usual control group. For the comparison of the standardized intervention with the self-tailored interventions, we expect a small to moderate effect (Cohen *d*=0.30) in favor of the self-tailored version. Power analyses for a repeated measures ANOVA with a within-between interaction for 3 groups in Switzerland estimated a sample size of at least 85 participants. For the 2-arm designs in Portugal and the Netherlands, we anticipate a dropout rate of 15% and, consequently, efforts will be made to recruit more primary end users than indicated by the power analysis.

For the target group of primary end users, older mourners, the aim is to include at least 85 participants in the study conducted in Switzerland with an allocation of 35:35:15 for the 2 active arms and the waitlist control group, respectively; at least 80 participants in Portugal with an allocation of 40:40; and at least 40 participants in the Netherlands, with an allocation of 20:20.

### Recruitment

Recruitment will vary across implementation countries to ensure a recruitment method that is adjusted to the specific study design of each country, sample characteristics, and country culture. Potential participants from the general population will receive an invitation to register in the study via newspaper, social media, internet, pastoral care, social care, or personal contacts from health care professionals. For each implementation country, an assessment of the recruitment strategy will be conducted 1 month after the start of the trial to decide whether the recruitment contingency plan will be implemented.

### Randomization

All eligible participants are randomly assigned to the active arm of the study (including the 2 active arms, standardized or self-tailored intervention format for the trial in Switzerland) or to the control or care-as-usual groups, depending on the implementation country. Participants assigned to the active arms will receive a participant code that gives them access to the self-help program and will be asked to use it for 10 weeks. Participants in the waitlist control group will start using the program 10 weeks after randomization. In Portugal, participants in the care-as-usual group can use the program after 20 weeks of follow-up, if requested.

For the 3 trials, the block-wise randomization was performed by an external programmer not involved in the trial using a random allocation sequence. The trials are unblinded. In Switzerland, the participants register on the study website and fill out the baseline questionnaire. Then, a member of the study team conducts the screening telephone call and allocates the participants to the 3 arms based on randomization numbers provided by the external programmer. In the Netherlands and in Portugal, participants are directly contacted by the teams responsible for implementing the study. Contacts are made based on their own professional lists. In the 3 countries, participants receive the intervention link after their eligibility has been confirmed in a phone call or an individual interview, depending on the implementation country.

### Description of the Intervention

#### Content

**Figure 1:**
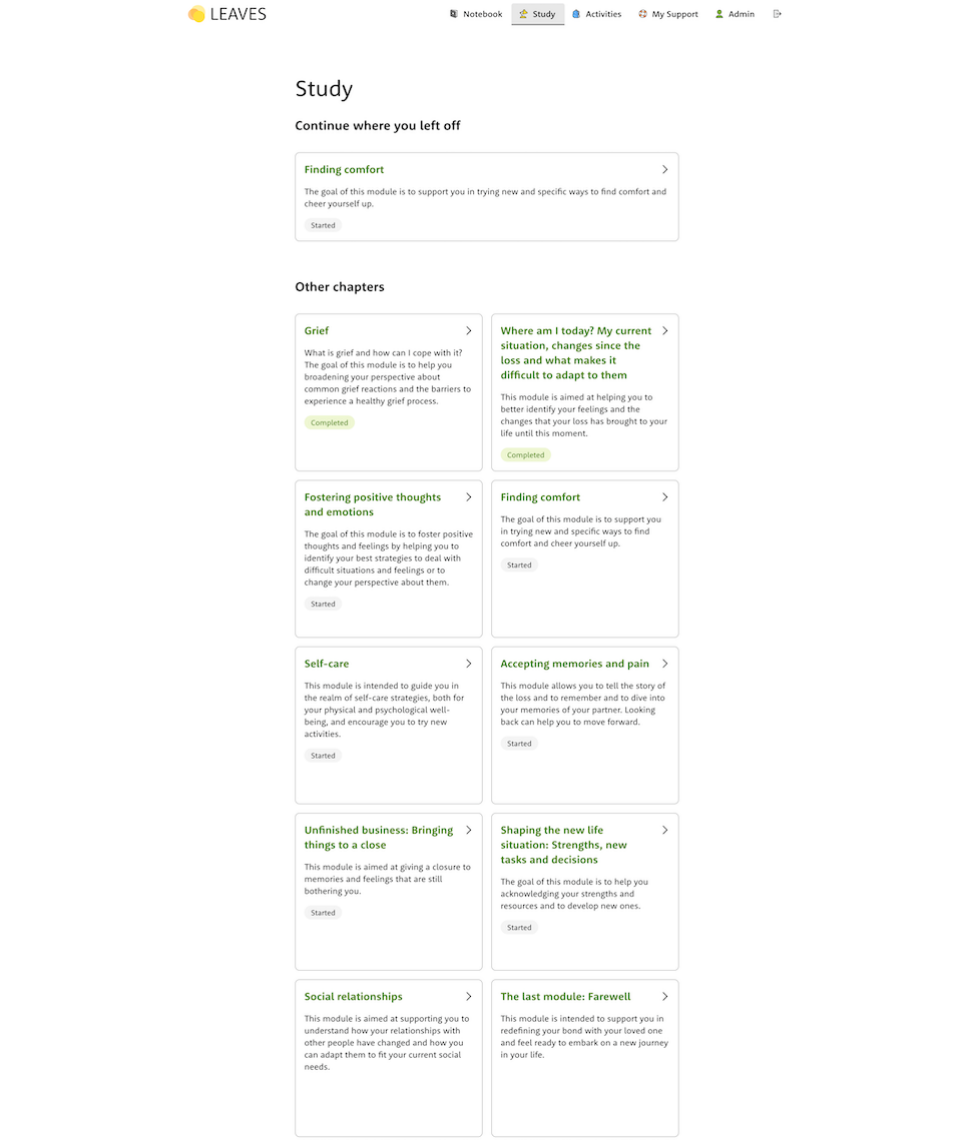
Screen of the web-based self-help program showing the first 6 modules and the available functionalities (right top corner).

The content of the intervention was designed to follow the content structure of LIVIA [14,34]. However, unlike LIVIA, the content will be provided to participants through a conversational virtual agent, Sun, who guides the user through the *Study* section (the CBT intervention itself; Figure 1). During the onboarding and the completion of study modules, users will be invited to use the other sections of the intervention: Notebook, Activities, and My Support. The Notebook is where users can find their notes from the study modules and are provided with a shortcut for the most important exercises. The Activities section provides suggestions for activities that encourage mourners to try new daily tasks or routines aimed at promoting mental and physical well-being. Finally, in My Support, participants can find a reminder of the hotlines or people they can contact to ask for additional support, alongside a summary of their best strategies to find support, which corresponds to some of the exercises completed in the study modules.

The main section of the intervention is the *Study* section which comprises 10 modules that address topics intended to support either the acceptance of the loss or the restoration and adaptation to a new life. The modules are divided in several submodules, which correspond to specific subtopics or different types of exercises. The first 2 study modules include general information about (1) the impact of the loss of a partner and grief reactions and (2) an assessment of the current personal situation. Modules 3 to 5 focus on resources and restoration-oriented tasks for fostering positive thoughts and emotions as well as self-care. Modules 6 and 7 consist of loss-oriented interventions for accepting memories and pain and for addressing unfinished businesses, events that the mourner may see as unresolved. Modules 8 and 9 again include restoration-oriented tasks focusing on creating a new life without the partner and social relationships. The last module, module 10, addresses the redefinition of the relationship with the deceased person.

The modules and submodules include (1) readings—that is, texts based on scientific knowledge about grief-related topics that provide the background and rationale for the modules; and (2) exercises encouraging mourners to actively reflect on their lives, their grief, and what was learned in the readings, besides applying their new knowledge to their daily life and regularly practicing the new coping strategies and routines. Within the *Study* section, the conversational agent introduces the modules and submodules, guides users through them, wraps up the modules and submodules, and suggests the completion of the self-reflection items, where participants are asked to assess how helpful the completed submodule was.

The content is delivered in 2 formats, corresponding to the 2 active arms of the trial conducted in Switzerland. In the standardized intervention format, the presentation of the modules is based on a fixed order, in which the next modules are only accessible after the former was completed. In the self-tailored intervention, only modules 1 and 2, focusing on information about grief reactions and an assessment of the current personal situation, respectively, are mandatory and are required to be completed at the start. The remaining modules become available after the completion of module 2, and from then on, the participants can decide on which module they want to work on next.

The German content of LIVIA was translated and further developed in English by the clinical team of the project. Subsequently, all content was translated from English to German, Dutch, and Portuguese by the research teams of each country with expertise in adult health care. The translated content was implemented in the web-based platform and tested by primary end users (older mourners) in user-experience tests. On the basis of the test results, changes were made in the English version by the clinical team of the project, followed by the respective translations. This iterative process of content development, translation, and testing ensured that the content is consistent among the implementation countries and is adapted to each cultural language specificities, without losing its clinical validity.

#### Mood Self-check

Participants will be asked every other week to complete a set of questions to self-check their mood and perceived progress in the program. This will also enable the program to assess whether participants are experiencing intense psychological distress. In addition, based on users’ answers in this self-check questionnaire, participants can be suggested to seek further support if high and prolonged distress in the mood self-assessment is reported or if they do not perceive that any progress was made while using the self-help program.

#### Participants’ Guidance During the Trial

During the 10 weeks of the intervention, users will be able to receive guidance, which varies depending on the country-specific research goal, target population, and structure of the research team. In Switzerland, participants will receive via email a short weekly feedback and support from a trained coach, whereas users in Portugal will be contacted via telephone every other week on the grounds of technical support and troubleshooting. This guidance is intended to acknowledge and motivate participants for their work with the self-help program and to provide a weekly structure for the use of the program. Moreover, it will ensure the identification of potential technical problems and create the opportunity for participants to ask further questions. This guidance will also be based on participants’ self-reported mood and therapeutic progress within the program. In the Netherlands, where the complementary research goal is to assess technology acceptance, guidance will follow a passive format in which participants will be able to contact the intervention’s team via email or telephone in case they need further support.

### Measures

#### Overview and Measurement Times

All clinical outcomes are self-report measures and will be completed on the web or within the program. In the trials conducted in Switzerland and the Netherlands, for the active arms, measurements of primary and secondary outcomes will take place (1) before users start using the program at baseline; (2) after the intervention, 10 weeks after the start of the program; and (3) at follow-up, 20 weeks after the user starts the program, which will measure the stability of the outcomes over time. For the waitlist control groups, the measurement times will occur at baseline (week 0), after 10 weeks waiting to start the intervention, after completing the intervention (week 20), and at follow-up (week 30). In the trial conducted in Portugal, the active arm and control group, care as usual, will occur at baseline, 10 weeks after starting the intervention, and at follow-up, 20 weeks after starting the intervention.

As no cross-cultural comparisons are planned, measures will be adapted to each country’s trial. The measures used in the 3 different countries depend on the measures available for each language, which will result in the use of equivalent measures when it is not possible to use the same. Primary and secondary outcomes will allow for testing the efficacy.

#### Primary Outcome: Grief Symptoms

In the 3 implementation countries, grief will be assessed with TRIG (eg, [37]). TRIG is a widely used measure to assess the severity of grief. A recent factor analysis identified 3 factors for emotional response, thoughts, and nonacceptance regarding a loss. TRIG assesses the severity of grief from 1=completely true to 5=completely false.

#### Secondary Outcomes

##### Depression Symptoms

In the 3 implementation countries, depression symptoms will be assessed by the Patient Health Questionnaire-9, a self-administered version of the Primary Care Evaluation of Mental Disorders diagnostic instrument for common mental disorders. The Patient Health Questionnaire-9 scores each of the 9 diagnostic criteria for major depression in the Diagnostic and Statistical Manual, Fourth Edition. For each item, the answer format is a scale from 0=not at all to 3=nearly every day [38].

##### Perceived Loneliness

Perceived loneliness will be measured with the de Jong Gierveld Loneliness Scale [39,40] in Switzerland and the Netherlands. These will use the revised and shortened version of the scale with 6 items that resolve into social and emotional subscales [41]. In the Portuguese trial, perceived loneliness will be measured by the University of California, Los Angeles Loneliness Scale [42]. This scale has 18 items that measure subjective feelings of loneliness and social isolation.

In the trials conducted in Switzerland and in the Netherlands, primary and secondary outcomes will be determined after the intervention and after 10 weeks of using the intervention for the active arms and after 10 weeks of waiting to use the intervention for the control group. In Portugal, primary and secondary outcomes will be determined after 10 weeks of starting the trial, for both the active arm and the care-as-usual groups.

#### Potential Mediators and Change Mechanisms

##### Working Alliance

In the Swiss trial, the working alliance will be measured by the Working Alliance Inventory (WAI) [43,44], which is a measure of the therapeutic alliance, a key variable that accounts for treatment outcomes and users’ satisfaction across interventions (eg, [45]). The items of the WAI adapted for guided internet interventions were derived from the WAI‐short revised [46] and adapted to internet intervention programs with therapist support [44,47,48]. The task and goals subscale will be the one used, as it measures agreement with tasks and goals of the program (therapy), which includes 12 items to be rated on a 5‐point scale, from 1=never to 5=always. The working alliance will be assessed at weeks 2, 5, 8, and 10 after starting the intervention.

##### Session Outcomes: Self-reflection Survey

In the 3 implementation countries, session outcomes, as possible change mechanisms, will be measured by a questionnaire with a 5-item short version based on the Bern Patient Questionnaire [49]. This measure includes the rating of the overall helpfulness of the module and the matching of participant’s needs and presented content, as well as gains in self-esteem, mastery experiences, and insight. Items are rated on a scale ranging from 3=not at all to 3=yes, exactly. Participants will be asked to complete the session outcomes items (self-reflection survey) after the completion of each submodule or module. In addition, these items related to the whole intervention will be assessed after the intervention.

#### Mood Monitoring

In the 3 implementation countries, mood monitoring will be measured by the mood self-check tool developed for this intervention, which assesses grief and depression symptoms, social withdrawal, and perceived progress within the intervention. Starting from the beginning of the web-based self-help intervention, the program will trigger an automatic message every other week asking participants to complete the mood self-check. Although this mood self-check is not part of the evaluation, it will inform the guidance of participants.

#### User Satisfaction

In the 3 implementation countries, user satisfaction will be measured by the Patient Satisfaction Questionnaire adapted for web-based interventions [50,51]. The Patient Satisfaction Questionnaire is a self‐report measure that explores patients’ overall satisfaction with the treatment. It includes 8 items that are rated on a 4‐point scale from 1=low satisfaction to 4=high satisfaction.

#### Use Data

Adherence will be measured through use, which will consist of the number of log-ins, completion of modules and submodules, and visits to the program’s pages. Use data will be assessed continuously while using the self-help program.

#### Context Measures and Sociodemographic Variables Assessed at Baseline

Context and predictor variables include age, gender, education, nationality, marital status, duration of relationship until the loss, time since the loss, and details about the loss (death due to illness, violence, suicide, or accident). These data will be collected in the 3 implementation countries.

#### Technology Acceptance

The trial conducted in the Netherlands is aimed at thoroughly investigating the technology acceptance of the intervention, specifically focusing on perceived usefulness (self-devised) and effort expectancy (based on the study by De Veer et al [52]), measured at baseline. The usability (based on the study by Holden [53]) and user experience using the AttrakDiff instrument (User Interface Design GmbH) [54] will be assessed midintervention. Finally, after the intervention (week 10), usability and user experience will be measured again, and participants will be asked to complete a measure of acceptance (self-devised) and effort (based on the study by De Veer et al [52]). In addition, at this phase, we will add open questions to assess the use and appreciation of mood self-check monitoring and respective feedback messages and critical incidents. As a follow-up, focus groups will be conducted to better understand the perceived benefit of the web-based self-help program. Focus groups will address usability and technical acceptance, answer which intervention elements need improvement, and assess the potential use of the intervention in daily and working routines.

The research team agreed to share all the materials used in the study, including the scales used (if open access) and the focus group scripts upon request to the corresponding author.

### Data Collection, Management, and Analyses

Data will be assessed using web-based questionnaires programmed in REDCap (Research Electronic Data Capture; Vanderbilt University) [55,56] or Qualtrics (Qualtrics) [57]. Data integrity will be enforced according to referential data rules, valid values, range checks, and consistency checks. Checks are applied at the time of data entry into a specific field. In addition, data on the use of the self-help sessions are collected within the platform. All data will be saved in an anonymous manner, only identified by a code that is not related to the participant’s identity. Data will be divided over 3 databases, an anonymized database containing data entered by the user into the platform (eg, exercises), an anonymized database containing use data logs, and a database that contains personal identifiers and the link to the internal anonymized identifier. After the end of the study, the last database will be deleted so the complete data set is anonymous. The platform and all data will be stored on International Organization for Standardization 27001–certified servers and will make use of secured connections. All data will be treated according to the guidelines of Dutch law and good clinical practices of the Swiss Federal Act on Research Involving Human Beings. Only the researchers directly involved in the study will have access to the data. Finally, it is important to note that this research collects data that allow identification of severe distress or mental health symptoms, including suicidal ideation. Following the public health guidelines on the management of personal data collection, this intervention is designed to flag the participants who show suicidal ideation or severe mental health crises that can be addressed in the guidance.

### Statistical Analyses

Dropout is defined as participants who withdraw actively from the study after randomization or fail to fill out the postintervention questionnaires despite 2 reminders. For technology acceptance, noncompliance is defined as not filling out the biweekly mood self-check questionnaire despite 2 reminders. Noncompliant participants are also defined as those who, despite not actively quitting the study, do not complete the 2 mandatory study modules (module 1 and module 2) within 10 weeks. Nevertheless, these participants will be a part of the intention-to-treat analysis, as they have been randomized. Adherence will be assessed with the number of modules completed and use data.

All rating scales will be checked for reliability (Cronbach α) and multicollinearity (correlation analysis). Results will be reported on scale averages, as well as multiple 2-way correlations. Analyses will be conducted according to the intention-to-treat paradigm. First, we will analyze the extent of missing data, explore the missing data patterns, and determine the type of missing data (missing completely at random, missing at random, or not missing at random). If the missing mechanism is missing at random, multilevel mixed effects regression analyses will be used, which allow a different number of measurement points per participant and are thus less sensitive to missing data.

Multilevel mixed effects models with repeated measures data will be conducted to evaluate the efficacy of the intervention after 10 weeks and the stability of the effects after a further 10 weeks. Restricted maximum likelihood estimation will be used, which is recommended for small group samples and yields asymptotically efficient estimators for balanced and unbalanced designs. Mixed effects models have several advantages. These consider the dependency of the data and account for the correlation of the repeated measures within individuals. Furthermore, mixed effects models use all available data of each participant and estimate parameters of missing values. Models for each outcome variable will be computed. The pre-post comparisons of all outcome measures will include time as a within-group variable, the intervention format as a between-group variable, and an interaction term time by a group for cross-level interactions. To test the stability of the effects from the postintervention period to follow-up, only time will be included as a within-group factor in the mixed effects models. Cohen *d* will be calculated as effect size for all observed outcome variables. Furthermore, a Reliable Change Index will be computed as a measure of clinical change. Complementary analyses will test whether variables such as age, time since loss, and the severity of grief symptoms have an effect on the outcome by including them as predictors in the regression models. To analyze the longitudinal interplay of predictor variables and test mediation analyses, structural equation models will be conducted. Analyses will be conducted using SPSS Statistics (IBM) [58] and Mplus [59] software.

To explain technology acceptance, a backward regression analysis will be conducted. All qualitative data will be analyzed thematically, following the guidelines by Braun and Clarke [60], whereas reporting will be done according to the consolidated criteria for reporting qualitative research standards [61].

### Cost-effectiveness Analysis

Incremental cost-effectiveness ratios of the intervention will be estimated, in terms of cost-per-point improvement in the grief scale (primary outcome) in the depression scale or in the loneliness scale (secondary outcomes), during the intervention and follow-up periods. A provider perspective will be adopted to mimic the *real-life*, posttrial, costs of the tool. Costs will include both technological (equipment costs and cost of using the platform) and human costs (training and time spent by staff delivering the intervention) associated with providing the intervention. To measure human costs, each coach will fill in a time sheet where they detail the number of minutes spent on each activity (eg, guidance or email reminders) for each user. Time is then multiplied by the hourly wage rate (including employer charges). To reflect statistical uncertainty (ie, sampling variation) with regard to both costs and effects, incremental cost-effectiveness ratio estimates will be accompanied by nonparametric bootstrapped SEs [62]. These analyses will be conducted separately by country, as the context and costs involved differ.

### Qualitative Evaluation of Implementation and Engagement

In Portugal, an additional qualitative study will be conducted to assess the barriers and facilitators to implementation, adoption, and engagement with the web-based self-help intervention. This study will be conducted separately with the primary and secondary end users (health care professionals) after the end of the intervention. Topic-guided interviews will be developed using the capability, opportunity, motivation, and behavior model [63] approach to assess barriers and facilitators in relation to aspects of competence, opportunity, and motivation to use the intervention. Thematic saturation will be used to determine the sample sizes, with a minimum of 10 interviews planned with each sample.

### Ethics Approval and Trial Registration

All participants will give written informed consent. In the 3 countries, a short telephone call will be conducted to assess whether participants meet the eligibility criteria. In Portugal, participants will have their eligibility fully checked in person, at baseline, with the presentation of the informed consent, after which the initial evaluation will continue. In Switzerland and Netherlands, eligible participants will receive a link to complete the primary and secondary outcomes for the baseline measurement.

Medical ethics approval has been obtained by the Medical Ethical Committee of Northwestern and Central Switzerland (Business Administration System for Ethics Committees number 2021-02221) and the Ethical Committee of Unidade Local de Saúde do Baixo Alentejo (EDOC/2021/48762). For the study in the Netherlands, the Medical Ethical Committee Oost-Nederland ruled that the study was exempt from obtaining medical ethical permission, as the main topic of the intervention and study (mourning) is not a medical condition (file numbers 2021-13268 and NL79937.091.21). The Portuguese and Swiss trials were registered at ClinicalTrials.gov (NCT05156346 and NCT05280041, respectively).

This study will be conducted in line with the Declaration of Helsinki, and no participant will be randomized unless written informed consent is available for that participant. Participants can withdraw from the trial at any time and will be informed and assured of such right. This study follows the principles of data protection and management described in the European Union’s General Data Protection Regulation. The trials were classified as having no or low risk; therefore, no protective measures for adverse events were mandatory. In the case of adverse events, the responsible ethics committees will be informed. Data monitoring committees are not required by the ethics committees. The ethics committees have the right to perform an audit at any time.

## Results

The trials started in March 2022 and are expected to end in December 2022 or when the needed sample size is achieved. The first results are expected by January 2023.

## Discussion

### Expected Findings

In 3 different trials, the present research expects to test the efficacy of a web-based self-help grief intervention for older mourners. Specifically, we expect that the intervention decreases grief symptoms, as well as depression symptoms and perceived loneliness after completing the intervention when compared with the control group. These outcomes are expected are to be stable at follow-up. Moreover, the trial in Switzerland will test the hypothesis that the self-tailored delivery format, by providing participants the opportunity to meet their specific needs at each moment, leads to better outcomes and to higher satisfaction with the intervention than the standardized intervention. In the trial conducted in the Netherlands, the results of this study will also provide insights into the acceptability of web-based self-help interventions directed at older adults who are affected by grief symptoms, psychological distress, or adjustment problems in daily life after the loss of their partner, as well as insights into the prevention of prolonged grief. Finally, in the trial conducted in Portugal, the cost-effective and qualitative analyses of the barriers to using the intervention, will provide insights on how to redirect the resources used to implement it.

The results extend the existing knowledge in several important areas. First, although most web-based grief interventions have focused on coping with prolonged grief or severe grief symptoms, this intervention also aims to support the mourning process after a more recent loss; for example, mourners who lost their partner at least 1 month before accessing the program. Therefore, it has a more inclusive target group than; for example, the LIVIA self-help program. Second, by comparing a standardized with a self-tailored intervention format, the study examines whether the efficacy of web-based grief interventions can be increased by providing a self-tailored version, in which the users can choose the content that seems most relevant to them and that fits their current needs best. This is in line with concurrent developments in face-to-face and web-based interventions [27], which explore the effects of personalizing interventions to the users’ specific needs. Third, the study aims to explore mediators of the effects of the intervention on the outcome; that is, the matching of participant’s needs and presented content, gains in self-esteem, mastery experiences, insights into one’s problems, as well as the working alliance.

Fourth, the study explores the acceptance of this technology among older adults. Technology acceptance among primary end users is crucial for the successful implementation of a web-based intervention in a real-life setting. A caring technology like this intervention should instill trust, should be engaging, and should provide a solid level of usability. To optimize the implementation of the web-based self-help intervention after the project phase, understanding technology acceptance is essential. Finally, a cost-effectiveness study complements the clinical research goals.

Furthermore, testing the implementation of this intervention in 3 countries will provide higher insights into the efficacy of the intervention in different contexts, enable specific analysis on the acceptability of the proposed technology, and provide further knowledge about the potential generalization of the web-based self-help intervention to a broader audience.

This research will ultimately contribute to meeting the health access needs exacerbated by the COVID-19 pandemic crisis. The development of an evidence-based self-help intervention on the web, dedicated to support older mourners’ mental health, will add to the efforts for equality and will contribute to reducing the stigma associated with mental health, death, and grief symptoms.

### Limitations and Future Directions

As a limitation, the self-selectivity of the sample may compromise the generalization of the results to a broad population of older adults, as older adults who are willing to take part in a web-based self-help intervention have more cognitive resources and a higher education level. The focus groups focused on technology acceptance will provide important insights into users’ skills and difficulties while using the self-help intervention, ultimately contributing to overcoming this potential limitation. Furthermore, we acknowledge that the meditation analysis may require a higher sample size to be informative and meet our research goals, and that, consequently, extending the sample size would be beneficial.

The adaptation of the designs of the trials to different complementary research goals and country-specific conditions, although it brings insightful knowledge, prevents cross-cultural statistical analysis and thus robust cross-cultural comparisons. To overcome this limitation, further research should test an established format of the intervention with the exact same study design. Although the present research brings relevant insights to the field of web-based self-help interventions, it leaves unanswered relevant questions. Despite this research advancing the knowledge on technology acceptance, future research based on this web-based self-help intervention could provide a direct test of the benefits of conversational agents and explicitly identify which of their components potentiate change. Moreover, it could be tested whether information on the grief process as well as the assessment of the personal situation have an impact on the outcomes by not making these modules mandatory in the self-tailored delivery format.

### Conclusions

To conclude, the 3 trials of the LEAVES project provide a comprehensive approach for advancing the knowledge on web-based self-help interventions for grief by integrating results on technology acceptance, barriers to and facilitators of using web-based self-help programs, predictors for the clinical efficacy, delivery formats including self-tailoring, as well as cost-effectiveness.

Finally, to foster the impact of this research, study results will be communicated by research peer-reviewed articles, conference talks, newspaper articles, and blog entries. Moreover, participants as well as the involved health care professionals will receive an email with a summary of the results at the end of the study. Importantly, one of the partners of the project will exploit the service, starting with the Dutch population. All the countries will actively contribute to the dissemination of the study protocol and their respective findings to bridge the gap between research and practice.

## Abbreviations

CBT: cognitive behavioral therapy
iCBT: web-based cognitive behavioral therapy interventions
LEAVES: Optimizing the Mental Health and Resilience of Older Adults That Have Lost Their Spouse via Blended, Online Therapy
REDCap: Research Electronic Data Capture
TRIG: Texas Revised Inventory of Grief
WAI: Working Alliance Inventory
